# Southern Surgical and Gynecological Association

**Published:** 1892-01

**Authors:** 


### SOUTHERN SURGICAL AND GYNECOLOGICAL ASSOCIATION.

### FOURTH ANNUAL MEETING HELD IN RICHMOND, VA., NOVEMBER 10, 11 AND 12, 1891.

NOVEMBER 11TH, SECOND DAY—MORNING SESSION.

The Association was called to order by the president at 10 a. m.

Prayer was offered by the Rev. Dr. Newton.

Dr. Thomas J. Moore, of Richmond, delivered an eloquent "Address of Welcome."

Dr. William Warren Potter, of Buffalo, New York, read a paper entitled "A Medico-Legal Aspect to Pelvic Inflammation," in which he said that pelvic inflammations in women have been described, discussed and debated from almost every point of view imaginable, until our periodical medical literature is flooded with articles on the subject, and medical society transactions are teeming and bristling with papers pertaining thereto. So far, however, he had not observed that anyone had undertaken to discuss these intra-pelvic conditions from a medico-legal standpoint. It was his purpose to present that aspect of the question, taking for his text a case that developed an interesting problem in that respect.

After giving a history of the case Dr. Potter emphasized the following points:

1. The intimate anatomical relations between the pelvic organs and the larger joints of the lower extremities, especially the hip and knee joints, render them liable to reflexes.
2. The importance of careful diagnosis at the outset, lest grave errors and possible disastrous consequences may result from treatment.
3. The medico-legal bearing that errors of judgment in diagnosis and treatment may have in relation to the patient, as well as upon the reputation of the physician.

"The Medico-Legal Aspect of Intestinal Surgery." Dr. John D. S. Davis, of Birmingham, Ala., read a paper on this subject. He said many physicians and surgeons who condemn all mechanical aids for intestinal repair, know not how to use them, never saw them used; refuse to endorse a resection for gunshot or stab wounds; have been known to go into the witness box for purposes of condemnation and disapproval when they knew no more about intestinal surgery than a wild Indian about school teaching.

In this day of specialties in medicine, but few general surgeons have either the appreciation, opportunity or disposition to qualify themselves as expert operators in intestinal surgery, but many—to the discredit of the profession—voluntarily appear in the criminal courts of the country pretending to be such—wise and proficient. One of the greatest professional sins of the day is perverted knowledge of conceited ignorance. It is too often that physicians and surgeons weaken and invalidate their opinions to a greater or less degree by unscrupulous interest in behalf of those employing them, a fact cunningly turned to advantage for defendants in criminal prosecution, and for like reason may become dangerous to the operators they oppose and envy.

To be able to do a laparatomy for stab or gunshot wounds of the intestines, inflicted by one with murderous intent, and be able to evade civil and criminal liability, the operator must (1) be able to show evidence of ordinary surgical knowledge in the requirement of the special operation to be performed; (2) he must possess ordinary surgical ability for doing the special operation to be performed; (3) he must exercise ordinary prudence in performing the special operation to be done, as to time, place, antiseptics, asepsis, assistance, nurses and after treatment; (4) he must perform the special operation in an ordinary skillful manner. Hence to prevent confusion it will be good, if possible to determine what constitutes ordinary surgical knowledge, ability, prudence and skill. Upon these depend the whole medico-legal status of the intestinal surgeon, and upon which the expert should be required to depend also. According to the practices and rulings of courts in this country, the word *ordinary* in its surgical adjectiveness, means that the surgeon shall be capable of and exercise that surgical knowledge, ability, prudence and skill with which a fair proportion of the surgeons of his given locality are endowed, and not that of the highest lights of his profession.

Dr. John A. Wyeth, of New York City, made some remarks on "Ether Anæsthesia."

Dr. Howard A. Kelly, of Baltimore, Md., followed with a paper entitled "Hand Disinfection."

Dr. I. S. Stone, of Washington, D. C., read a paper on the "Pedicle in Hysterectomy."

The principal methods were described and illustrated by colored drawings, showing the arrangement of the pedicle in the abdominal wound. The author claims a revival of interest in the operation, and that there is need for its frequent performance. The statistics are far better now than ovariotomy claimed after it had become an operation of election, and was firmly planted in public favor.

Particular attention is given by the author to tying off the broad ligaments and the use of the elastic ligature. Sewing the parietal peritoneum to that of the pedicle in the extra-peritoneal cases was also dealt upon.

The method by ventro-fixation had given good results in the author's hands, and served to accomplish two important purposes, viz: A speedy convalescence, and avoids the disagreeable sloughing which follows the use of the wire clamp. It may also be used in some cases of short pedicle where the wire may not easily be applied. The methods were compared and statistics furnished, showing that the extra peritoneal method with wire and pin gave better results than either of the others. That ventro-fixation came next and the intra-peritoneal method came last, with a large m rtality. A method of closing the capsule over the stump was described, which the author claimed would answer for either *dropping* it or sewing in the wound—ventro-fixation. In the latter case the suspensory sutures are placed and the pedicle sewed in and under the lower end of the abdominal incision. Great care is required in closing the capsule over the raw surface of the stump so that separation may not occur. Owing to the peculiar contractile nature of the capsule, care must be taken to leave sufficient length for approximation of peritoneal surface.

The uterine arteries are to be tied in any case when hemorrhage is likely to occur, and drainage may be required. Besides reference to methods the author described the process through which the wound passes subsequent to supra-vaginal hysterectomy.

All myomatous tissue should be removed, which can only be effected in some cases by a process of reduction of the pedicle. This is very important, as in the operation where a large amount of myoma is left, more time is required for atrophy and absorption to reduce the pedicle to its proper size. Great danger to the patient is apt to follow, where a broad base of the tumor is left in either method of treatment, because this mass must be disposed of before the patient entirely recovers.

The author had observed a sufficient number of cases to declare that permanent fixation of the stump to the abdominal wall was the rule, where the extra abdominal methods were used, and especially when the broad ligaments were cut away to prevent traction.

SECOND DAY—AFTERNOON SESSION.

The association was called to order by the First Vice-President, Dr. J. McFadden Gaston, of Atlanta, Georgia.

The first thing in order was the Annual Address of the President, by Dr. L. S. McMurtry, of Louisville, Ky. Dr. McMurtry selected for his subject "A Plea for Progressive Surgery." He said within fifteen years the entire practice of surgery has been revolutionized. New methods have been introduced and new regions invaded; comparatively recent teachings have become obsolete in practice, and modern treatises recast. The science and art of gynæcology, which a few years since was limited to a small and narrow field, has grown into a great branch of medical science and practice. Formerly divided between midwifery and surgery, as a minor branch of one or both, gynæcology has become an independent and essential department of the healing art.

When Marion Sims announced through the columns of the British Medical Journal that he believed the proper course of treatment in every case of gunshot wound of the abdomen is to open the stomach, search for the bleeding points and secure them, and suture intestinal perforations, he was pronounced by many eminent surgeons to be a dreamer. The suggestion of Sims was most timely, and shortly afterwards Bull successfully executed the operation. For years the treatment of opium in full doses had been pursued, with death in waiting. Now there is scarcely a State in the Union that one or more patients have not been rescued from certain death by prompt resort to operative treatment. He mentioned these circumstances to illustrate and emphasize the point that surgery is advanced more by the aggressiveness of the surgeon than by timidity. In the face of desperate conditions of disease and injury, where there can be no safety whatever in delay and palliation, the only treatment worthy of consideration is the aggressive course which promises success. Under such conditions the most heroic surgery is conservative, and any other course is not conservative.

Dr. Thomas Addis Emmet, of New York, read a paper entitled "Injuries to the Pelvic Floor and the Method of Repairing the same."

Dr. Joseph Taber Johnson, of Washington, D. C., followed with a paper on "The Growth of Fibroid Tumors of the Uterus after the Menopause."

He said the object of the paper was to put on record cases and opinions in opposition to this view of this important subject, and to aid in recasting our views and in modifying our practice.

He had within the last five years seen at least a dozen women with large growing and troublesome fibroid tumors of the uterus, who were over 50 years of age; some of them over sixty. These women had been assured by their physicians that if they could get along somehow until after the change of life their tumors would not only stop growing, but that they would lessen in size, and probably go away altogether; at least, the troublesome and dangerous symptoms would disappear. They had been advised against any radical operation, and encouraged to believe that as they grew older they would get entirely well. In perhaps the majority of cases this might prove to be very good advice, but the point which the author wishes to make is, that as we are now better acquainted with the history and behavior of these tumors, this is no longer safe advice to give. We cannot assure any woman that her tumor may not prove to be one of the exceptional cases, and that it may not grow more rapidly after the menopause than it did before, or that it may not present complications equally distressing and disastrous. When from forty to fifty per cent. of women subject to supra-vaginal hysterectomy died from the effects of the operation, this was very safe and conservative counsel to follow. The possible dangers of the tumor were not equal to the probable dangers of the operation.

The author drew the following conclusions:

1. That the "rule" stated in the text books that the uterine fibromata ceased to grow after the menopause, has many more exceptions than is generally supposed.

2. That when they continue to grow after the menopause they pursue a more disastrous course than before.

3. They more frequently become cystic, calcareous or have abscesses develop in them.

4. These conditions, requiring operation according to well-known rules of surgery, the patients are in a less favorable condition for recovery than before the menopause.

5. If the above conclusions are admitted to be true it must follow that they furnish additional indications for more frequent and earlier resort to the radical operation.

In the hands of the best operators in cases where a pedicle can be secured, the mortality of supra-vaginal hysterectomy is rapidly approaching that of ovariotomy.

"The Surgical Treatment of Anterior Displacements of the Uterus." Dr. C. A. L. Reed, of Cincinnati, Ohio, read a paper on this subject.

He said anterior displacements of the uterus, when they exist to the pathological degree, are the opprobria of the gynecic art. It is indeed true that many wombs lean far forward without inducing symptoms, but it is likewise true that many of them that are thus malposed do entail symptoms, objective and subjective, that frequently baffle our resources. It is a misfortune too, that many of all the displacements in which the womb is liable, those in which the organ deviates anteriorly to the normal axis are vastly the more prevalent. Thus in an aggregate of four hundred and ninety-four cases by Nonat, Meadows, Scanzoni, Valloix and Hewitt, quoted by Thomas and Munde, there were two hundred and ninety-four ante-flexions and one hundred and eighty retro-flexions; while Munde himself reports two hundred and ninety-four ante-flexions, thirty three retro-flexions, and ten latero-flexions in a total of three hundred and thirty-seven cases. As the latter authority is disposed to look upon ante-flexions in their minor stages as a physiological (even congential) condition, it is legitimate to infer that his statistics are based upon observations of displacements in the pathological degree. The conclusion is forced upon us then, that of all the displacements of the uterus, those of the anterior variety are the more frequent; while the records of practice will force us likewise to the conclusion that of all of the womb displacements those of the anterior variety are less amenable to treatment than are any of the others.

In the treatment, the term *surgical* is employed in contra-distinction to any method of treatment by pessaries, tamponnage or electricity. It may be premised that all surgical methods devised for the relief of these conditions should be directed, first, to the removal, when practicable, of the causes of the diseased conditions proper, and, finally, to the readjustment of the diseased organs to the normal physical forces of the pelvis.

In conclusion the author desired the Association to consider

1. The etiological relationship of contracture of the utero-sacral ligaments to ante-flexion.

2. The possibility of overcoming this condition by such conservative measures as rest, pelvic depletion and appropriate manipulations.

3. The feasibility of removing the obstructive dysmenorrhœa and the sterility usually incident to these cases by the plastic operation which he had described.

4. The inexpediency of forcible dilatation for the relief of these cases and its inability to effect a permanent cure.

"The Part the Shoulders play in Producing Laceration of the Perineum, with Suggestions for its Prevention." This was title of a paper read by Dr. W. D. Haggard, of Nashville, Tennessee, in which he made the following suggestions:

1. The patient should occupy the left lateral decubitus at least during the second stage of labor.

2. Overcome rigidity of the vulvar outlet by the judicious use of chloroform.

3. The presenting part of the child should be supported and not the perineum during the passage of the head and shoulders.

4. Support the head by pressing it well up under the symphysis pubis by placing the right thumb in the rectum and fingers of the right hand expanded over the occiput.

5. To retard the exit of the shoulders, pressure should be applied to the trunk and shoulder by placing the index and middle fingers of the left hand in the rectum with the thumb in the vagina to restrain its exit.

6. Support the head and neck by pressure well over the symphysis pubis.

---

NOVEMBER 12TH, THIRD DAY—MORNING SESSION.

The Association was called to order by the President at 10 a. m.

Dr. James A. Goggans, of Alexander City, Ala., read a paper entitled "Abdominal Section in a Case of Cyst of the Mesentery." He stated that he had been induced to write a paper on the case from the fact that cysts of the mesentery are extremely rare, and that operations for their removal are most generally fatal. He said that he had been able to find the record of one case of cyst of the mesentery removed by enucleation by Guyon. The patient died on the 7th day after the operation. One case operated upon by Sir Spencer Wells, the operator in that case incised and drained the cyst, but the patient died within a few weeks. Three cases operated upon by Pean, only one of which recovered. One case operated upon by Watts, but that he did not know the result in the case. One case operated upon by Cortes, who incised and drained the cyst, but the patient died from septicæmia and hemorrhage. One case operated upon by Bantock, who removed the cyst by enucleation and the patient recovered.

The conclusion arrived at as to the origin of the cyst in that case, both by Dr. Bantock and the pathologist who examined the specimen, was that it originated from some fœtal structure, possibly some of the rudiments of the permanent kidney. He said that Dr. Greig Smith says, that he knows of two cases of mesenteric cyst removed by operation by his friend, but that he could not relate them to him, as they had not yet been published. He said that the patient upon whom he had operated for a cyst of the mesentery was a young woman, 21 years of age, daughter of a physician of Columbus, Ga. She had not been well for two years, but did not know that her abdomen was becoming larger until three months before the operation. During those three months she had been treated for abdominal dropsy, and had suffered much uneasiness and pain in the abdomen, and at the time of the operation her pulse was 120, and the temperature 100 degrees Fahr. The cyst was quite large, occupied mostly the left side of the abdomen, extended from under the ribs into the left lumbar region, dipped downward into the pelvis and extended three or four inches beyond the median line of the abdomen into the right side. He said that he first removed about a quart of the fluid by aspiration on February 7, 1891. The fluid was thin and of a dark color, and contained albumin, phosphate and chlorides. The patient was not benefited by the operation, and the abdominal section for the removal of the cyst was made on February 24th, 1891.

The cyst was covered with omentum and mesentery, and loops of small intestines were imbedded in its walls. An attempt was made to enucleate it, but hemorrhage was so free that the idea of enucleation was soon abandoned. A point as remote as possible from blood vessels and intestines was selected, the cyst incised and drained. More than one gallon of a thin, dark-colored fluid was evacuated, the sac irrigated with hot water, the lips of the incised sac stitched to the upper angle of the abdominal incision, and a glass drainage tube introduced to the bottom of the cyst. The abdominal incision was then closed with silk worm gut sutures. The author was confident that the cyst was retro-peritoneal. The time consumed in the operation was 25 minutes. The sac was irrigated three or four times in the twenty-four hours, and the drainage tube gradually withdrawn. The patient suffered much from nausea and vomiting, which he attributed to the close connection between the walls of the sac and the loops of small intestines. The patient made a good recovery within thirty days. He presented a picture of the patient which was taken the 1st of November, 1891, which showed her to be in perfect health.

"Thinness of Uterine Walls Simulating Extra-Uterine Pregnancy, With Report of Two Cases," was the title of a paper by Dr. Geo. J. Englemann, of St. Louis, Mo.

The author said there are many difficulties in the way of a positive diagnosis of early pregnancy, even in cases surrounded by conditions less unusual, but they assume alarming proportions when aggravated by the curious complications which may arise in individual cases, and above all when conditions are simulated in which delay is dangerous and operative interference seems called for, when a decision is urgently demanded, a decision upon which a life, and perhaps two, may depend. Whilst the auditor may criticize at his leisure and readily differentiate the conditions depicted, it is only he who is to pronounce and to act who can realize the difficulties of this entangling and so knotty a problem.

CASE 1—Patient 32 years of age, had borne three children in the six and a half years of her married life, the youngest twenty months ago, which was still nursing, and the menstrual flow has not as yet reappeared since the birth of this child. The patient came to his clinic for relief from a variety of discomforts from which she had been suffering more or less for the past three months. She complains of sick headache, vomiting spells, fullness of the stomach, belching after meals, and intermittent swelling of the abdomen, a pain in the groin appearing before such swelling, and a small tumor above the right groin, which she first noticed three weeks ago, and as she stated, "then suddenly made its appearance." An examination revealed large varicose veins over the lower limbs; a solid, round, movable tumor above symphysis and right groin; the cervix low and large; the uterine body thickened, lying low in the pelvis, with a certain mobility independent of the super-imposed tumor; an applicator entering three and a half inches slightly ante. Notwithstanding the wine color of the pronounced cystocele and the cervix, pregnancy seemed out of the question, and the tumor was diagnosed as most probably a dermoid of the right ovary, hardly one connected with the uterine wall. In the course of an examination two weeks later, a very different condition of affairs was revealed. The tumor had disappeared and a fœtus was found in the utero-vesical space, freely movable, apparently floating about, the small parts being distinctly felt as if underneath a wet towel both through the vagina and abdominal walls. So distinct did the small parts appear to the examining finger, that it seemed impossible to realize that even as much as a thickness of the vaginal tissues should intervene, and the abdominal walls must certainly have been very much attentuated to disclose the fœtal parts with such distinctness. Probe showed the uterine cavity free six and a half inches in length, still slightly ante, but never curving forward in the direction of the previous tumor.

The treatment for the supposed subinvolution was discontinued, the patient warned to keep quiet and to notify Dr. Englemann upon the occurrence of any abnormal symptoms. He believed the case to be one of ectopic gestation, either within the broad ligament or in the abdominal cavity after tubal rupture marked by the sudden appearance of the tumor five weeks ago, yet he was not sufficiently positive to warrant the immediate resort to the knife, and well that he did not do so, as persistent treatment and repeated examinations resulted in labor pains and the delivery of a five months fœtus in the most correct and natural manner.

Dr. Robert T. Morris, of New York, contributed a paper on "The Removal of Necrotic and Careous Bone with Hydrochloric Acid and Pepsin."

The author said sometimes it is desirable to remove dead bone without subjecting a weak patient to a dangerous or deforming operation. Attempts have been made with some success at clearing out this bone by a process of defalcification, but there are two chief reasons why failures have resulted as a rule. In the first place, it was discovered that superficial layers of dead bone were decalcified easily enough, but the acids did not reach deeply through the mass, especially if portions were infiltrated with caseous or fatty debris. In the second place, cellulitis was pretty apt to develop during the course of treatment.

After much experimentation he had finally adopted a method of work which seemed to be complete. An opening is made through soft parts by the most direct route to the seat of dead bone, and if sinuses are present, they are all led into the one large sinus, if possible. The large direct siuts is kept open with antiseptic gauze and the wound is kept open with anni septic gauze and the wound allowed to remain quiet until granulations have formed. Granulation tissue contains no lymphatics, and absorption of septic material though it is so slow that we have very good protection against cellulitis. The next step consists in injecting into the sinus a two or three per cent. solution of hydrochloric acid in distilled water. If the patient is confined to bed the injections can be made at intervals of two hours during the day; but if it is best to keep the patient up and about, the acid solution is thrown into the sinus only at bed time. In either case the patient is to assume a position favorable for the retention of the fluid. Decalcification of exposed layers of dead bone takes place quickly, and then comes the necessity for another and very important step in the process. At intervals of about two days an acidulated pepsin solution is thrown into the sinus (he uses distilled water, oz. iv, hydrochloric acid, m. xlv; Fairchilds' pepsin, dr. ss.) and this will digest out decalcified bone and caseous and fatty debris in about two hours, leaving clean dead bone exposed for a repetition of the proceedure. The treatment is continued until the sinus closes from the bottom, showing that the dead bone is all out.

Even in distinctly tuberculous cases the sinuses will close if apparatus for immobilizing diseased parts and tonic constitutional treatment are employed, as they should be, in conjunction with our efforts at removing the dead bone. If suppuration is free in any cavity in which we are at work, it is well to make a continual practice of washing out the cavity with peroxide of hydrogen before each injection.

It is a popular impression that living bone is not attacked by dilute mineral acids, but as it makes a good deal of difference whether the impression is correct or not, he experimented as follows: A portion of the keratinoid layer was removed from the carapace of a turtle (nanemys guttatus) and the animal was then placed tail downward in a glass of five per cent. hydrochloric acid solution. In the glass he placed also a segment snipped from the plastron of the turtle, and a transverse segment from an old dry humerus of a man. The piece of humerus was completely decalcified in six hours; the segment from the plastron was soft in about twenty hours; and the carapace of living bone was decalcified at the exposed part in thirty hours. He was then curious to know what effect the acid had had upon the blood vessels of the decalcified bone, and Dr. Smith, of the laboratory section of the Post-Graduate Medical School, made for him several sections of the carapace which included both decalcified and healthy bone. Investigation showed that all of the blood vessels were destroyed wherever the bone was softened, and the action of the acid had extended farther up along the larger blood vessels than elsewhere.

The difference in time between decalcification of the dead bone—six hours, and of living bone, thirty hours, is significant; a five per cent. solution of the hydrochloric acid having been used. If we use a two or three per cent. solution of hydrochloric acid a wall of lymph and of granulation tissue is thrown out upon the surface of the living bone for protection, and only dead bone is attacked. This at least has been his observation in several cases in which the results of treatment could be easily watched.

Dr. Landon Carter Gray, of New York, in a paper entitled "The Present Status of Cerebral Surgery," touched upon the modern aspect of intra-cranial surgery. The speaker first passed in review our present knowledge of localization of functions of the brain, stating that we were well acquainted with the functions of the motor area, of the third frontal convolutions, the cuneus, certain portions of the basil ganglia, the base of the brain and the cerebellum, and that we know nothing, or had still under discussion, the localization of the centers for the sensations of touch, pain, muscular sense, temperature sense, most of the parietal lobe and most of the temporo-asphenoidal lobe with the exception of the olfactory lobe. He stated that operations for fracture of the skull with or without hemorrhage, for abscess and for tumors that were removable and localizable, were usually successful; those for so-called idiopathic epilepsy were utterly valueless, as were also those for epilepsy supposed to be due to genital or ovarian irritations, whilst those done for epilepsy due to removable or localizable lesions of the intra-cranial contents were usually successful so far as the lesion was concerned, although it was a grave question as to whether the epileptic habit was ever cured; the latest operation for idiocy supposed to be due to premature ossification of the fontanelles was still under discussion and consideration, the cases being too few and too recent to permit of any conclusion; whilst the operation for hydrocephalus and for epilepsy due to such early infantile and fœtal lesions as parencephalus, hemorrhage and meningitis, were indefensible. He further impressed upon surgeons the great difficulty that there often was in finding a sub-cortical lesion of the centrum ovale that was deep-seated or small, and the fact should be borne in mind that there might be no decussation of the motor fibres from the hemispheres, so that a lesion would be found upon the same side as the paralysis.

"Some Complications of Psoas Abscess." This was the title of a paper read by Dr. J. McF. Gaston, of Atlanta, Ga.

---

THIRD DAY—AFTERNOON SESSION.

Dr. Paul B. Barringer, of Richmond, Va., read a paper on "Venomous Serpents of the United States and the Treatment of Wounds Inflicted by Them."

Dr. Christopher Tompkins, of Richmond, Va., followed with a paper entitled "A Case of Induced Abortion for Relief of Nausea and Vomiting, with Remarks."

On August 1, 1885, he was called to see Mrs. J., aged 24, and, as nearly as could be ascertained, three and a half months pregnant with her first child. Patient was born in the mountainous part of Virginia; she had an active outdoor life and grew up to be a woman of good height and of round full figure. January 14, 1884, she was married. While in the city of New Orleans, in stepping from the platform of a car, she sprained her ankle. This, although treated immediately by a physician of that place, and subsequently in this city, caused her great suffering. Finally, refusing to yield to the usual treatment, the part was put in a plaster cast; she went about on crutches, and after many months recovered. In the meantime she became pregnant, and from the first was attacked with nausea and vomiting. Mild in the beginning, it gradually increased in gravity, till she sent for him on the 1st of August, 1885. Her husband stated that she had had fever for two weeks. He found her in bed and learned that she had been there for days; her figure not robust and her face thin and attentuated. What little she had eaten in the past ten days or two weeks had been apparently rejected, her temperature one degree above normal; tongue foul; sordes on the teeth and the breath of a sour and bilious odor. The pulse was fairly good, considering her condition. Even the mention of food was distressing to her, and the sound of the dinner bell, though far off from her, caused so much distress, that its ringing was discontinued by the family. The bowels throughout her pregnancy had been constipated, only moving once in two or three days. Although continuously retching, very little or no blood had been seen in the material vomited, except on two occasions, and then not a great deal, and such as there was, was of a florid, scarlet color. No medicine had been given, and no treatment taken except the occasional use of lime water, which she said "did no good."

The patient did not improve up to August 7th, when Dr. Tompkins, thinking the case one of the greatest gravity, and that the question of abortion could no longer be deferred, invited Drs. J. B. McCaw and Aaron Jeffery to meet him in the afternoon in consultation. All agreed that aboition must be produced, in order to give the patient a last chance for her life, which was done.

REMARKS: The case is reported principally because it was an unsuccessful one, and because he wished to disabuse the minds of those who are not experienced in such operations of the notion, commonly believed, and often expressed, that the induction of abortion for the nausea and vomiting of pregnancy is, in skillful hands, an undertaking devoid of danger and necessarily attended by success. In this case, he is of the opinion that death was the result of the protracted debility and enfeebled constitution, due to long confinement and suffering; first, from the injury to her ankle, from which she had not recovered when she became pregnant, and was attacked by nausea and vomiting, this last continuing until her death. Under such circumstances the outlook was indeed very unfavorable, for to the shock of operation and depression incident to the use of chloroform, there was added fever and protracted prostration, both from injury to the ankle and from want of nutrition, the result of the long existing nausea and vomiting. He had before and since operated on women for the nausea and vomiting of pregnancy and with success, whose apparent condition was much worse than that described in the above case, but without the history of a previous injury or disease.

The prognosis, always unfavorable, ought, when the case is so complicated, to be of the most guarded kind. The practitioner should not, however, hold his hands on this account, for the operation affords the poor sufferer the only opportunity of relief. The author uses metal dilators instead of tents, and completes the operation at one sitting. He is likewise convinced that the least possible chloroform used, the better the result.

The following officers were elected:

President—Dr. J. McFadden Gaston, Atlanta, Ga.

First Vice-President—Dr. Cornelius Kollock, Cheraw, S. C.

Second Vice-President—Dr. Geo. Ben Johnston, Richmond, Va.

Secretary—Dr. W. E. B. Davis, Birmingham, Ala.

Place of next meeting, Louisville, Ky., second Tuesday in November, 1892.

Chairman of Committee of Arrangements—Dr. L. S. McMurtry, Louisville, Ky.

